# Athlete Experiences of Shame and Guilt: Initial Psychometric Properties of the Athletic Perceptions of Performance Scale Within Junior Elite Cricketers

**DOI:** 10.3389/fpsyg.2021.581914

**Published:** 2021-04-29

**Authors:** Simon M. Rice, Matt S. Treeby, Lisa Olive, Anna E. Saw, Alex Kountouris, Michael Lloyd, Greg Macleod, John W. Orchard, Peter Clarke, Kate Gwyther, Rosemary Purcell

**Affiliations:** ^1^Orygen, Parkville, VIC, Australia; ^2^Centre for Youth Mental Health, The University of Melbourne, Parkville, VIC, Australia; ^3^School of Psychology and Public Health, La Trobe University, Melbourne, VIC, Australia; ^4^School of Psychology, Deakin University, Melbourne, VIC, Australia; ^5^Cricket Australia, Melbourne, VIC, Australia; ^6^La Trobe Sport and Exercise Medicine Research Centre, La Trobe University, Melbourne, VIC, Australia; ^7^Specialist Sports Medicine Centre, Canberra, ACT, Australia; ^8^Faculty of Medicine and Health, University of Sydney, Sydney, NSW, Australia

**Keywords:** guilt, shame, self-conscious emotions, distress, mental health, help-seeking

## Abstract

Guilt and shame are self-conscious emotions with implications for mental health, social and occupational functioning, and the effectiveness of sports practice. To date, the assessment and role of athlete-specific guilt and shame has been under-researched. Reporting data from 174 junior elite cricketers (M = 17.34 years; females *n* = 85), the present study utilized exploratory factor analysis in validating the Athletic Perceptions of Performance Scale (APPS), assessing three distinct and statistically reliable factors: athletic shame-proneness, guilt-proneness, and no-concern. Conditional process analysis indicated that APPS shame-proneness mediated the relationship between general and athlete-specific distress (*p* < 0.01), with this pathway non-contingent on sex or past 12-month help-seeking for mental health concerns (*p*'s > 0.05). While APPS domains of guilt-proneness and no-concern were not significant mediators, they exhibited correlations in the expected direction with indices of psychological distress and well-being. The APPS may assist coaches and support staff identify players who may benefit from targeted interventions to reduce the likelihood of experiencing shame-prone states.

## Introduction

The ways in which athletes appraise their performance and the associated self-attributions can influence perceptions of athletic success or achievement, and the perceived need for reparative action where performance is viewed as suboptimal (e.g., below their known ability level). While some athletes may internalize a critical or harsh narrative to motivate enhanced training or preparation, this approach is typically counterproductive, with the potential for unfavorable comparisons relative to peers, past performance, or goal attainment (Powers et al., 2009) and the possibility of a loss of confidence after perceived failure (Stoeber et al., 2008). If the central goal of athletic coaching is to use the coach-athlete relationship to facilitate positive changes in athlete competence, confidence, connection and character (Côté and Gilbert, 2009; Vella et al., 2010), to effectively execute these responsibilities, coaches need access to a depth of information beyond performance and achievement metrics. Assessing, and where necessary ameliorating problematic athletic self-perception — especially in relation to negative self-conscious emotions (e.g., shame and guilt) — is likely to be an important aspect of facilitating domains of competence, confidence, connection and character that contribute to athlete well-being and performance. While maturation processes may support youth athletes developing insight into, and self-management practices for experiences of problematic self-conscious emotions in the sporting context, suitably supportive, and nurturing coaching environments may serve to bolster and enhance innate coping, and fast-track adaptive coping responses in relation to shame and guilt.

Shame and guilt are negative affective experiences that tend to occur following a performance-related failure, or a behavioral transgression, that is perceived as inappropriate, morally wrong, or below internally (or externally) prescribed standards (Tangney, 1991). Though shame and guilt are commonly experienced emotions, they are often (mistakenly) referred to interchangeably. Theorists distinguish these affective states based on the focal point of one's negative evaluation (Lewis, 1971; Tangney et al., 1996). Specifically, when shamed, the *self* becomes a focal point of negative scrutiny and the event that elicits the shame response is often internalized and attributed to stable character flaws (e.g., “*I* failed and therefore *I* am incompetent”; Lutwak et al., 1998; Tangney and Dearing, 2002). Conversely, with feelings of guilt the focus of the negative evaluation is squarely on the discrete regrettable behavior or event, rather than the self (e.g., “What I *did* was wrong”; Tangney et al., 1996). Guilt is also an empathy triggering other-oriented emotional process, in that the individual is acutely aware how their behavior adversely impacts others (e.g., “*I've let my team down*”; Treeby et al., 2016).

Guilt and shame are associated with different motivational and behavioral outcomes. When shamed, the self is interpreted as irreparably flawed and that little can be done to rectify it (Kaufman, 1989). In this sense, the motivational and behavioral outcomes associated with shame tend to be maladaptive and can include avoidance, withdrawal, and disengagement (Tangney, 1991). Alternatively, as experiences of guilt tend to be distinct from an individual's self-concept, the behavioral and motivational outcomes are typically adaptive, as they promote a reparative response (e.g., wanting to cease the problematic behavior or fix the wrongdoing). In comparison to guilt, shame is a much more aversive and disabling experience implicated in a larger range of negative psychological outcomes including motivation and goal striving (Weiner, 1985, 1986; Tangney and Fischer, 1995). Shame-proneness has also been linked with maladaptive perfectionism, depression, distress, anxiety, and substance use as a means of coping with negative emotions (Derogatis et al., 1973; Cook, 1996; Thompson et al., 2003; Treeby and Bruno, 2012).

At present, there is limited research into the experiences of athlete shame and guilt, and there are no sports-specific screening tools for athletic shame and guilt. Research from alternative achievement-based settings (e.g., university) has demonstrated that highly shame-prone individuals that experienced a perceived performance failure were less likely to put effort into similar subsequent tasks (Thompson et al., 2003). Therefore, it may follow that if an athlete experiences shame due to a suboptimal performance (in competition or in training), this athlete may feel less inclined to train harder through increased practice effort, may fail to remain engaged in similar achievement related tasks (e.g., a competitive match), and fail to set task-related goals (e.g., increasing training load). As a direct result of task-related disengagement and a lack of goal-striving behavior, there may also be a reduction in task-related performance (e.g., Thompson et al., 2003). Shame-proneness in particular has been associated with lower mental toughness among athletes, although self-forgiveness was found to mediate this relationship — hence, being mentally tough may actually signify the tendency and/or ability to be more forgiving of one's athletic shortcomings (Cowden et al., 2018). This finding is supported by the growing evidence base of self-compassion focussed interventions among athletes (Mosewich et al., 2019; Wilson et al., 2019), given this approach is known to reduce shame-proneness and associated mental health symptoms (Gilbert and Procter, 2006; Johnson and O'Brien, 2013).

Though research regarding athlete shame and guilt is in its infancy, the implications on future achievement motivation, mental health and well-being, and potential dysfunctional self-protective behaviors is made clear in the broader literature (Tangney and Dearing, 2002; Thompson et al., 2003; Hofseth et al., 2015). As elite sports research is moving toward holistic understanding of athlete psychological and physical health, well-being and performance (Purcell et al., 2019), exploring the degree and impact of shame and guilt in athletes is paramount to informing this global picture. To best understand these concepts, a primary need for validated and athlete-specific measurement tools exists. Extant scales that measure guilt and shame typically use a trait-based approach measured using scenario based items where the respondent is asked how they would react in a given transgressional situation (e.g., the Test of Self-Conscious Affect (TOSCA); Tangney et al., 2000). While widely validated in the general population, these hypothetical situations (e.g., social or moral transgressions) are less directly relevant to the athletic and sporting achievement settings. As argued by Mills (2005), there is a need for domain and context-specific measures of self-conscious emotions. Similarly, existing achievement-based scales, for example, the Achievement Guilt and Shame Scale (AGSS; Thompson et al., 2008) still utilize hypothetical scenarios that will not necessarily reflect experiences of perceived sporting failure.

Given the preliminary nature of sporting guilt and shame literature, and the necessity for domain specific measurement tools, the purpose of this study was to develop and undertake initial psychometric validation of a domain-specific measure of athlete guilt and shame. Identification of sport-specific self-conscious emotions, and their mental health and well-being correlates, may support enhanced targeted early intervention programs in the future. In a sample of elite junior cricket players, we expected exploratory factor analysis to support the existence of distinct putative athlete guilt- and shame-proneness factors, correlating negatively with psychological well-being and positively with general and athlete-specific psychological distress, with higher observed guilt- and shame-proneness female athletes as per existing literature (Else-Quest et al., 2012). Further, athlete shame-proneness in particular was expected to account for additional variance (via mediation analysis) in the relationship between general psychological distress predicting to athlete-specific distress.

## Methods

### Participants

Australian junior cricket players attending either the male U19 National Championships or the female U18 National Championships were invited to participate. Survey data were provided by 174 players (males *n* = 89, females *n* = 85), with a mean age of 17.34 years (*SD* = 1.00).

### Measures

#### Demographic Data

Non-identifying demographic information was collected.

#### Athlete Psychological Strain Questionnaire (APSQ)

The APSQ is a brief 10-item screening tool for athlete mental health, which has been shown to have acceptable validity in male (α = 0.87) and female (α = 0.84) elite athletes (Rice et al., 2019, 2020a). The APSQ includes three subscales assessing self-regulation (e.g., “*I was irritable, angry, or aggressive”*), performance concerns (e.g., “*I found training more stressful”*), and external coping (e.g., “*I needed alcohol or other substances to relax*”) in addition to a scale total score. Responses are measured on a five-point Likert scale from *1* = *none of the time* to 5 = *all of the time*.

#### Kessler Psychological Distress Scale (K10)

The K10 is a 10-item screening tool to assess psychological distress, such as nervousness, fatigue, hopelessness, and depression (Kessler et al., 2003). This tool has been widely validated in a range of populations (Donker et al., 2009; Cornelius et al., 2013; Bougie et al., 2016) including elite athletes (males α= 0.86, females α = 0.80; Rice et al., 2020a). The scale relates to the previous 4 weeks, and responses are measured on a five-point Likert scale where *1* = *none of the time* and 5 = *all of the time*.

#### Warwick-Edinburgh Mental Well-Being Scale (WEMWBS)

The WEMWBS is a 14-item scale assessing positive aspects of mental health as a single factor, such as feeling useful, relaxed, and optimistic (Stewart-Brown et al., 2009). Responses are measured on a five-point Likert scale where *1* = *none of the time*, and *5* = *all of the time*. The scale has also been validated with elite athletes (males α = 0.94, females α = 0.93; Rice et al., 2020a).

### Scale Development – The Athletic Perceptions of Performance Scale

The Athletic Perceptions of Performance Scale (APPS) was purposively designed to be a brief measurement tool to fill an existing gap in the assessment of athlete-specific self-conscious emotions relative to performance, namely, athletic shame- and guilt-proneness, and no performance concerns, assessed over the past 4 weeks. Following review of the theoretical literature related to the role of self-conscious emotions in achievement-related settings authors MT, SMR, LO, and RP collaboratively developed an initial item set assessing domains of athletic shame and guilt-proneness. The initial item pool was subsequently shared with researchers and practitioners based in the elite setting, who provided expert feedback on wording, clarity and item construction. Following this, a series of item iterations were undertaken until an item pool of 12-items was finalized, which notionally comprised three domains (each with four items), assessing (i) athletic guilt-proneness, with items focusing on the need for reparative performance-based actions (e.g., “*I felt a need to train harder for future matches/contests”*), (ii) shame-proneness, with items focussing on a perceived defective athletic self-identity (e.g., “*I felt useless as a player/athlete”*), and (iii) no-concern, with items focussing on no perceived performance issues (e.g., “*I had no performance issues to worry about”*). The no-concern items were developed to identify those athletes who perceived that they were performing well and were satisfied with their efforts. These items were included to ensure that the scale was relevant to all athletes, irrespective as to how positively or negatively they appraise their performance. Respondents completed the APPS after reading the following introduction “*These questions concern how you have felt following your overall performance over the past 30 days. Please select the answer that best represents your experience where 1 (Strongly disagree) to 5 (Strongly Agree).”*

### Procedure

High performance managers notified staff (e.g., coaches, team managers), players, and their parents/guardians in the months prior to the age group National Championships of the survey. Parents/guardians were encouraged to discuss participation with their child prior to them attending the Championships, however participants aged over 16 years were able to consent without parent/guardian approval. At the Championships, a member of the research team presented to each team and invited players to complete the online survey after reading the participant information statement. Players were advised that participation was voluntary and that their decision to participate or individual data would not be identifiable. A psychologist was present at the time of survey completion and throughout the Championships, and details of additional external support (either online and phone) were also provided. The survey was administered via a secure online platform and participants completed the survey on their own mobile device. The average time for survey completion was under 10 min (mean = 9 min, 47 s). Ethics approval was granted by the La Trobe University Human Research Ethics Committee (HEC19480).

### Data Analysis

Descriptive statistics were calculated for all demographic variables to characterize the sample. Between-groups analyses (*t*-tests, χ^2^) tested for sex differences. Scale internal consistency values were evaluated using Cronbach's coefficient. In order to identify the number of factors to retain for the APPS, parallel analysis was undertaken using the SPSS macro rawpar.sps (O'Connor, 2000). Parallel analysis is one of the most accurate factor retention methods, providing more reliable factor solutions compared to traditional methods of evaluating scree plots and Eigenvalues >1 (Hayton et al., 2004). Following parallel analysis, principal axis factoring was undertaken, reporting the Kaiser-Meyer-Olkin (KMO) Test for Sampling Adequacy (where KMO ≥ 0.70 = good; Hair et al., 2006) and Bartlett's test of sphericity. Direct oblimin rotation was used to enable identified factors to correlate. Per scale development guidelines (Stevens, 1992), any scale items with factor loadings below 0.40 were deleted, as were any items cross-loading >0.32 (DeVellis, 2016). Analyses were re-run following deletion of any items and the final rotated pattern matrix was inspected to guide factor identification and interpretation. Divergent validity was examined by non-parametric (Spearman's) correlations between APPS domains and the WEMWBS (negative associations expected between the APPS guilt- and shame-proneness domains and WEBWBS total score). Convergent validity was assessed by Spearman correlations (reported separately by gender) between APPS domains and APSQ and K10 (positive correlations expected between the APPS guilt- and shame-proneness domains and the APSQ and K10). The APPS no concern domain was expected to be unrelated to the well-being indices (e.g., no statistically significant correlations observed with the APSQ, K10, or WEMWBS). Mediation analysis was undertaken using the PROCESS macro (Hayes, 2017) to determine the role of WEMWBS and APPS domain scores in moderating the K10 – APSQ relationship. A secondary conditional process analysis was undertaken to determine whether observed mediation effects were contingent on sex or past 12-month mental health help-seeking. Separate parallel bootstrapped models were evaluated (normal distribution not required), using 99% CIs and 10,000 bootstrap resamples using PROCESS models 4 and 16 (see Hayes, 2017). In these models, K10 scores (*x*) predicted to APSQ scores (*y*), evaluating APPS domains as parallel mediators (*m*), and moderators participant sex (*w*) and past 12-month help seeking (*z*). Analysis of APPS quartile distribution explored corresponding categories of psychological distress. All analyses were undertaken in SPSS 26.0.

## Results

The response rate for the eligible population participating at the Championships was 62% for males (89/143), and 77% for females (85/111). Male participants (*M* = 17.93 years, *SD* = 0.84 years) were significantly older than female participants (*M* = 16.73 years, *SD* = 0.75 years), *p* < 0.001. See Table 1 for participant demographics.

**Table 1:** Participant demographics.

	**Male (*n* = 89)**	**Female (*n* = 85)**
**Cultural and ethnic background**		
Australian	85	78
Indigenous Australian or Torres Strait Islander	<5	7
New Zealander	<5	<5
African	<5	<5
Asian	<5	5
Indian	7	<5
European	<5	<5
**Studying**		
Secondary (high school)	30	64
Tertiary (university)	20	17
Certificate or diploma	<5	-
Trade or apprenticeship	10	-
No	27	<5
**Involvement with cricket in last month**		
Regularly playing/training	87	77
Irregularly playing/training	-	<5
Restricted playing/training due to injury/illness	<5	6
Restricted playing/training due to other commitments	-	<5
**History of psychological treatment**		
Yes, in the past 12 months	14	10
Yes, not in the past 12 months	2	5
No	73	70

Parallel analysis was undertaken with the APPS item pool, yielding three underlying factors within the dataset. The factorability of the data was “good” (KMO = 0.761) and Bartlett's test of sphericity was significant (*p* < 0.001). Principal axis factoring with direct oblimin rotation was undertaken, with a specified three factor solution. The three factors accounted for 53.29% of scale variance, and were consistent with the theoretically aligned constructs of shame-proneness (eigenvalue 3.98; 30.30% of variance), guilt-proneness (eigenvalue 2.01; 12.84% of variance), and no-concern (eigenvalue 1.62; 10.16% of variance). All items reported factor loadings >0.40 with the expectation of a single shame-proneness item “*I found it hard to face my teammates or coach*.” Due to the low loading, this item was omitted. The analysis was re-run (KMO = 0.754, Bartlett's test *p* < 0.001), with the three factors accounting for 56.84% of scale variance, consistent with the initial analysis of shame-proneness (three items; eigenvalue 3.86; 32.07% of variance), guilt-proneness (four items; eigenvalue 1.98; 13.73% of variance), and no-concern (four items; eigenvalue 1.64; 11.05% of variance). There were no cross-loading items >0.32. The rotated factor solution is presented in Table 2.

**Table 2:** APPS descriptive statistics and factor loadings.

**APPS item**	***M (SD)***	**Response frequency % (*****n*****)**					**Factor loadings**		
		**1 “*Strongly disagree”***	**2**	**3**	**4**	**5 “*Strongly agree”***	**Shame-proneness**	**Guilt-proneness**	**No- concern**
*I felt useless as a player*	2.40 (1.00)	18.1 (31)	41.5 (71)	24.6 (42)	14.0 (24)	1.8 (3)	**0.932**	−0.046	0.037
*I felt worthless and not good enough as a player*	2.45 (1.01)	17.0 (29)	38.6 (66)	28.7 (49)	12.9 (22)	2.9 (5)	**0.895**	−0.047	−0.007
*I felt like I'm a poor player*	2.48 (0.91)	11.2 (19)	45.3 (77)	29.4 (50)	12.4 (21)	1.8 (3)	**0.880**	−0.060	−0.024
*I felt a need to work harder for my team*	3.55 (0.96)	1.8 (3)	14.1 (24)	24.7 (42)	45.9 (78)	13.5 (23)	−0.012	**−0.692**	0.077
*I felt a need to train harder for future matches/contests*	3.63 (0.97)	1.2 (2)	14.0 (24)	22.8 (39)	44.4 (76)	17.5 (30)	0.126	**−0.630**	−0.119
*I felt a need to remove distractions, so I could focus*	3.16 (0.92)	2.4 (4)	23.5 (40)	33.5 (57)	36.5 (62)	4.1 (7)	0.007	**−0.568**	0.070
*I realized I need to avoid repeating mistakes*	3.54 (0.91)	2.9 (5)	9.4 (16)	29.2 (50)	48.0 (82)	10.5 (18)	0.040	**−0.491**	−0.182
*I saw no problems with my performance*	2.32 (0.89)	17.1 (29)	44.7 (76)	28.8 (49)	8.2 (14)	1.2 (2)	0.155	0.179	**0.796**
*I felt that I performed my best*	2.98 (0.93)	5.3 (9)	24.7 (42)	39.4 (67)	27.6 (47)	2.9 (5)	−0.164	−0.140	**0.719**
*I had no performance issues to worry about*	2.19 (0.82)	18.3 (31)	50.9 (86)	24.9 (42)	5.3 (9)	0.6 (1)	0.063	0.097	**0.654**
*I felt that I performed well*	3.18 (0.85)	2.9 (5)	17.1 (29)	41.8 (71)	35.3 (60)	2.9 (5)	−0.246	−0.157	**0.596**

*Bolded text indicates grouping of each factor*.

Internal consistency values for the three APSS domains were all satisfactory (APPS Shame-proneness α = 0.94; APPS Guilt-proneness α = 0.71; APPS No-concern α = 0.79), as were the Cronbach coefficients for the K10 (α = 0.89), WEMWBS (α = 0.90), and APSQ with the exception of the external coping subscale, which reported marginal reliability in the present sample (Self-regulation α = 0.77; Performance concerns α = 0.77; External coping α = 0.55; APSQ total score α = 0.85).

APPS subscale means and SDs were evaluated by sex (see Table 3). Small effects for participant sex were observed for the APPS Shame-proneness and APPS Guilt-proneness domains, in addition to the K10 and WEMWBS, however only the effect for APPS Shame-proneness survived correction for multiple comparisons (*p* < 0.01). Female respondents reported higher scores relative to males, with the exception of the WEMWBS where males scored higher.

**Table 3:** APPS means and SDs by sex.

	**Total**	**Male**	**Female**	**Inferential**	**Effect size**
	***M (SD)***	***M (SD)***	***M (SD)***	***t*, *p***	**Cohen's *d***
APPS Shame-proneness	7.36 (2.75)	6.78 (2.63)	7.96 (2.75)	−2.87,0.005	0.41
APPS Guilt-proneness	13.90 (2.74)	13.48 (2.66)	14.33 (2.77)	−2.06,0.041	0.31
APPS No-concern	10.67 (2.75)	10.71 (2.99)	10.63 (2.48)	0.20,0.845	0.03
K10	18.81 (6.54)	17.82 (6.13)	19.85 (6.82)	−2.05,0.042	0.31
WEMWBS	48.21 (7.64)	49.45 (6.81)	46.92 (8.26)	2.19,0.030	0.33

Domain associations (Spearman correlations) are reported in Table 4, with associations ranging from weak to strong, with negative correlations observed for variable pairings with the APPS No Concern domain, and the WEMWBS. Bonferroni adjusted *r* to *z* transformations indicated that correlations did not significantly differ by sex.

**Table 4:** Correlations (Spearman) between constructs and domains by sex.

	**1**.	**2**.	**3**.	**4**.	**5**.	**6**.	**7**.	**8**.	**9**.
1. APPS Shame	−	0.18	−0.51^***^	0.39^***^	0.47^***^	0.33^**^	0.49^***^	0.47^***^	−0.54^***^
2. APPS Guilt	0.13	−	−0.32^**^	0.15	0.24^*^	0.17	0.21	0.26^*^	−0.21
3. APPS No concern	−0.24^*^	−0.12	−	−0.31^**^	−0.40^***^	−0.24^*^	−0.40^***^	−0.46^***^	0.52^***^
4. APSQ Self-regulation	0.41^***^	0.10	−0.15	−	0.62^***^	0.41^***^	0.87^***^	0.77^***^	−0.53^***^
5. APSQ Performance concerns	0.44^***^	0.35^**^	−0.20	0.57^***^	−	0.42^***^	0.91^***^	0.67^***^	−0.36^***^
6. APSQ External coping	0.17	−0.02	0.01	0.40^***^	0.18	−	0.52^***^	0.40^***^	−0.27^*^
7. APSQ Total	0.49^***^	0.26^*^	−0.22^*^	0.86^***^	0.88^***^	0.42^***^	−	0.79^***^	−0.50^***^
8. K10	0.49^***^	0.06	−0.08	0.72^***^	0.52^***^	0.43^***^	0.73^***^	−	−0.58^***^
9. WEMWBS	−0.33^**^	0.14	0.29^**^	−0.23^*^	−0.22^*^	−0.08	−0.25^*^	−0.38^***^	−

*Females above diagonal, males below diagonal*.

* *p < 0.05*,

** *p < 0.01*,

*** *p < 0.001*.

Partial correlations were calculated between APPS Shame-proneness and APPS Guilt-proneness with the APSQ, K10, and WEMWBS. As construct associations reported in Table 4 did not differ, analyses were not sex disaggregated. Table 5 shows the previously significant correlation between guilt proneness and K10 (*r*_s_ = 0.18, *p* = 0.014) scores was no longer significant (*r*_*s*_= 0.13, *p* = 0.106), indicating no relationship between APPS Guilt-proneness and psychological distress when controlling for APPS Shame-proneness perceptions of performance.

**Table 5:** Partial correlations (Spearman) for APPS Shame- and Guilt-proneness.

	**Shame-proneness partial**^**a**^	**Guilt-proneness partial**^**b**^
APSQ Self-regulation	0.39^***^	0.09
APSQ Performance	0.45^***^	0.23^***^
APSQ External coping	0.23^**^	0.03
APSQ Total	0.48^***^	0.21^**^
K10	0.47^***^	0.13
WEMWBS	−0.44^***^	0.41

a *Controlling APPS Guilt-proneness*;

b *Controlling APPS Shame-proneness*.

** *p < 0.01*,

*** *p < 0.001*.

Mediation analysis inspecting non-overlapping 99% CIs indicated that APPS Shame-proneness (β = 0.099, SE = 0.033, 99% CI 0.023–0.192) significantly mediated the relationship between K10 predicting to APSQ scores (total effect predicting to APSQ: *F*_(1,166)_ = 276.77, *p* < 0.001, *R*^2^ = 0.625). Neither APPS Guilt-proneness (β =0.017, SE = 0.012, 99% CI −0.004 to 0.058), APPS No-concern (β = 0.005, SE = 0.014, 99% CI −0.040 to 0.043), or WEMWBS scores (β = −0.010, SE = 0.032, 99% CI −0.097 to 0.074) were significant mediators. Conditional process analysis indicted the APPS Shame-proneness mediation effect was not contingent on gender (β = −0.040, SE = 0.057, 99% CI −0.115 to 0.181), or past 12-month mental health help seeking (β = −0.017, SE = 0.324, 99% CI −0.255 to 0.247). In summary, mediation modeling indicated the effect of K10 on APSQ scores occurred via APPS Shame-proneness, and that this effect was not contingent on gender or recent mental health help-seeking status.

Quartiles for the APPS Shame-proneness scale were examined (see Table 6). Players in the fourth quartile (APPS Shame-proneness ≥9) were on average in the “Very high” range for the APSQ, and “High” range for the K10, indicative of the need for coaching and/or mental health intervention. Quartile group comparisons for the APSQ Shame-proneness scale with adjusted *post-hoc* analysis (Scheffe) indicated that each APPS quartile group differed from the others (all quartile comparison *p*'s < 0.001), with a large effect size *F*_(3,166)_ = 362.79, *p* < 0.001, partial *?*^2^ = 0.855. For the K10, adjusted *post-hoc* analysis indicated that three of the six quartile group comparisons differed from the others (quartiles 1 and 3 *p* = 0.025; quartiles 1 and 4 *p* < 0.001; quartiles 2 and 4 *p* = 0.003), with a large effect size *F*_(3,166)_ = 11.92, *p* < 0.001, partial η^2^ = 0.177.

**Table 6:** APPS Quartiles and corresponding APSQ and K10 indices.

**APPS Shame-proneness quartile**	**APPS Shame-proneness score**	**APSQ**		**K10**	
		***M* (SD)**	**Category descriptor**	***M* (SD)**	**Category descriptor**
1st	3–5	14.00 (3.36)	“*Normal”*	14.53 (4.02)	“*Normal”*
2nd	6	15.98 (3.93)	“*Moderate”*	17.65 (5.50)	“*Moderate”*
3rd	7–8	18.00 (4.36)	“*High”*	19.23 (5.83)	“*Moderate”*
4th	≥9	20.86 (6.79)	“*Very high”*	22.07 (7.21)	“*High”*

## Discussion

As hypothesized, distinct factors were validated for the APPS Shame- and Guilt-proneness subscales, in addition to a distinct no-concern factor, which was negatively associated with both. The higher observed ratings of shame- and guilt-proneness among female players are consistent with findings observed in the general community (Else-Quest et al., 2012). While distinct and statistically unrelated to each other (e.g., non-significant Spearman's correlations) the APPS Shame- and Guilt-Proneness subscales both demonstrated moderate positive associations with general psychological distress and athlete-specific distress (as assessed by the K10 and APSQ respectfully) and were inversely related to psychological well-being. Given those in the uppermost quartile of the APPS Shame-proneness subscale were also, on average, classified in the high distress range on other measures, the shame-proneness domain may have particular utility in identifying players that may benefit from coaching, clinical and/or performance psychology intervention.

As indicated, both APPS Shame- and Guilt-proneness were positively associated with concerns regarding one's performance and issues relating to selection pressures, concerns regarding injury, and training related stress. However, when controlling for guilt-proneness, only APPS shame-proneness was positively associated with a range of clearly maladaptive self-regulatory outcomes in the elite sport context. Indeed, associations with the APSQ items indicated that shame-proneness was associated with the self-reported tendency to engage in risk taking behavior, the use of substances to cope, issues with irritability and aggression, reduced motivation, and detachment from one's teammates. Discrete experiences of performance related guilt were unrelated to these same problematic self-regulatory and coping strategy outcomes. These findings provide support for the external validity of the APPS, and suggest that athletic performance related shame may be associated with a host of negative sequela for young athletes.

Findings from the conditional process analysis indicated that shame-proneness, but not guilt-proneness, mediated the relationship between general and athlete-specific distress, and that this relationship was not moderated by participant sex or recent mental health help seeking. When interpreted in the light of other findings reported above, this finding (while preliminary and requiring replication) suggests that the effect of athlete shame-proneness is not driven by mental ill health or the observed sex difference in shame, but rather explains unique variance in the relationship between general distress and psychological distress. These findings are underscored given the stringent use of 99% CIs, and the evaluation of parallel mediators (e.g., guilt-proneness and well-being). Consistent with previous literature (e.g., Lutwak et al., 1998) and theory (e.g., Tangney and Dearing, 2002), this highlights the particularly aversive and impactful nature of shame, and warrants both further empirical study and exploration of intervention targets.

In achievement settings, feelings of shame arise when an individual fails to adequately perform a task, and attributes this failure to perceived global incompetence (Weiner, 1985, 1986). Athletes are socially regulated by an array of internally prescribed standards (i.e., from the self, coaches, teammates), and externally prescribed standards (i.e., from the public, media, and social media). In this regard, there is ample opportunity for perceived performance failure among athletes. Given athletic identity is largely based on performance success, shame may be induced by sports performance failure or athletic inability (Lazarus, 2000; Conroy, 2004), especially in shame-prone individuals. Broader assessment of the APPS domains would inform prevalence of athletes experiencing these affective states, and could provide coaching and sports medicine professionals with a tool that aids identification of athletes that may be at risk of experiencing maladaptive failure reactions.

A practical extension of the present study would be examining APPS domains in the context of brief sport-specific interventions, and situations of maladaptive coping to avoid shame states in particular. One pertinent example is athlete self-handicapping, where an athlete may present oneself with a hindrance or barrier to performance, which is perceived to reduce chances of success or achievement (Snyder, 1990). While self-handicapping is typically perceived as a transgression according to social and moral codes within sports performance, and may elicit guilt feelings (Munroe et al., 1999). Hofseth et al. (2015) found that in elite soccer players, shame-proneness had a direct positive relationship to behavioral self-handicapping, and guilt-proneness had a direct negative relationship to behavioral self-handicapping. Longitudinal studies could look to explore the temporal associations between athlete shame and guilt, self-handicapping, performance and other key variables, including the coach-athlete relationship, and other indices of mental ill health including substance misuse.

Regarding potential intervention, self-compassion-focussed therapies are gaining increasing interest in the sports medicine context (e.g., Mosewich et al., 2019; Walton et al., 2020). Self-compassion approaches seek to develop athlete abilities to engage with distress in a compassionate manner to activate affiliative processing systems, and brief measures of the construct exist, which may be useful in assessing self-compassion in the sports setting (e.g., Raes et al., 2011; Steindl et al., 2021). Previous research has found that self-compassion is negatively correlated with self-criticism (*r* = −0.61) and positively correlated with perceived sport performance (*r* = 0.29; Killham et al., 2018), hence the development of self-compassion skills may reduce the likelihood of experiencing shame. There is emerging evidence (in non-elite settings) that suggests coaching and high-performance staff, including sports psychologists, should build team awareness of how team-based norms of self-compassion evolve, particularly given greater perceived self-compassion within teams is associated with higher *individual* self-compassion (Crozier et al., 2019). As such, investment in focussed professional development for coaching and high-performance staff to enhance team-based cultures of self-compassion (while simultaneously balancing the rigors and expectations of elite performance), may support environments where athletes can gain insights and coping strategies to support their mental skill development in parallel with sporting skills.

The present findings need to be considered alongside several important limitations. The validation sample reported in the present study was comparatively small and lacked diversity. As the sample consisted of junior elite cricket athletes, future research with the APPS is needed across a wider range of sporting disciplines, in addition to testing wider psychometric properties of the scale (e.g., differential item functioning) across salient demographic groups such as culture, age, education, and socioeconomic status. Future work should also consider person-centered approaches to assessing change in athlete APPS scores over time, such as latent growth curve modeling (e.g., Rice et al., 2020b). Also, from a sport-specific perspective, cricketers' experience significantly more day to day performance fluctuation attributable to luck compared to many other sports (Bhanushali and Bagchi, 2020). This means that the cricket context and associated luck (or more specifically, bad luck) may result in cricket players being more likely to question their own abilities in comparison to other athletes, which may in turn influence perceptions of shame and guilt. Accordingly, we call for additional validation of the APPS across representative and diverse athlete populations globally. Such cross-cultural validation efforts are underway with the Sports Mental Health Assessment Tool (SMHAT-1) from the International Olympic Committee (Gouttebarge et al., 2021) and the APSQ (Rice et al., 2020b) used in the present study, which, like the APPS, provide bespoke athlete-centered psychological assessment tools. While this study demonstrated associations between the APPS domains and indices of positive and negative mental health functioning, the implications for indictors of athletic performance and other related variables (e.g., team functioning, depth of coach-athlete relationship, motivation) are unclear, and an important line of future inquiry. Further, data was collected from junior-level elite athletes, and generalization of these findings to senior players or athletes are uncertain. A highlighted above, longitudinal research is required as the present data is cross-sectional in nature, prohibiting analysis of temporal factors. The present study was nested in a larger piece of research, and as such, we were limited in the number of validated scales that could be utilized. The consequence of this is the limited convergent and divergent validity information is available for the APPS domains, and further research using established measures of guilt and shame (e.g., TOSCA, Tangney et al., 2000; PFQ-2; Rice et al., 2018) is needed to explore overlap between the APPS and other widely used scales and domains. Finally, there is an urgent need for the development and evaluation of athlete-specific mental health interventions. Notwithstanding some notable exceptions (e.g., Donohue et al., 2018) randomized controlled trials are needed. While there is growing knowledge related to the unique mental health challenges that elite athlete experience and new individual-based models are being developed and implemented (Rice et al., 2020c), relatively little is known from the existing literature of controlled trials regarding the best type and form of team-based intervention. While self-compassion focussed therapies appear promising, high quality trials are needed.

In conclusion, the present study offers the field a new tool for assessing athlete-specific guilt and shame. While this initial validation study provides robust data on the factor structure of the APPS, more work is required to demonstrate the clinical or performance utility of the scale. Nonetheless, given guilt and particularly shame are known to exert problematic consequences in non-sporting achievement contexts, they are also likely to impact domains of athletic achievement, and thus warrant further applied research.

## Data Availability Statement

The datasets presented in this article are not readily available because we do not have ethics approval to make the data available open access.

## Ethics Statement

The studies involving human participants were reviewed and approved by La Trobe University Human Research Ethics Committee (HEC19480). Written informed consent for participation was not provided by the participants' legal guardians/next of kin because Completing the Athletic Perceptions of Performance Scale was deemed low risk. Parents/guardians were encouraged to discuss participation with their child, however participants aged over 16 years were able to consent without parent/guardian approval. A psychologist was present at the time of survey completion, and details of additional external support (either online and phone) were also provided.

## Author Contributions

SR was responsible for data analysis and preparation of the manuscript, and played a role in design of the study. MT, LO, KG, and RP contributed significantly to data interpretation and write-up of the manuscript. AS coordinated the study, managed logistics and approvals, and assisted with data interpretation. AK, ML, GM, JO, and PC supported study conceptualization, facilitated data collected, and assisted with ethics approval and post-approval processes. All authors have reviewed and approved the final manuscript.

## Conflict of Interest

The authors declare that this study received in-kind funding from Cricket Australia. Representatives from the funder had the following involvement in the study: Assistance with data collection, decision to publish and preparation of the manuscript.
